# TrkC Intracellular Signalling in the Brain Fear Network During the Formation of a Contextual Fear Memory

**DOI:** 10.1007/s12035-023-03292-0

**Published:** 2023-03-08

**Authors:** Francisca Silva, Gianluca Masella, Maria Francisca Madeira, Carlos B. Duarte, Mónica Santos

**Affiliations:** 1 CNC – Center for Neuroscience and Cell Biology, University of Coimbra, Coimbra, Portugal; 2 Institute of Interdisciplinary Research, University of Coimbra (iiiUC), Coimbra, Portugal; 3 Department of Life Sciences, University of Coimbra, Coimbra, Portugal

**Keywords:** Fear conditioning, Neurotrophin 3, Erk, Akt, PLC

## Abstract

Learned fear is orchestrated by a brain fear network that comprises the amygdala, hippocampus and the medial prefrontal cortex. Synaptic plasticity within this network is critical for the formation of proper fear memories. Known for their role in the promotion of synaptic plasticity, neurotrophins position as obvious candidates in the regulation of fear processes. Indeed, recent evidence from our laboratory and others associates dysregulated signalling through neurotrophin-3 and its receptor TrkC with the pathophysiology of anxiety and fear-related disorders. Here, we put wild-type C57Bl/6J mice through a contextual fear conditioning paradigm in order to characterize TrkC activation and expression in the main brain regions involved in (learned) fear – amygdala, hippocampus, and prefrontal cortex – during the formation of a fear memory. We report an overall decreased activation of TrkC in the fear network during fear consolidation and reconsolidation. During reconsolidation, hippocampal TrkC downregulation was accompanied by a decrease in the expression and activation of Erk, a critical signalling pathway in fear conditioning. Moreover, we did not find evidence that the observed decrease of TrkC activation was caused by altered expression of dominant negative form of TrkC, neurotrophin-3, or the PTP1B phosphatase. Our results indicate hippocampal TrkC inactivation through Erk signalling as a potential mechanism in the regulation of contextual fear memory formation.

**Supplementary Information:**

The online version contains supplementary material available at 10.1007/s12035-023-03292-0.

## Introduction

Excessive (learned) fear is a shared feature of anxiety disorders [1], the most prevalent group of mental disorders worldwide with more than 300 million people affected by one of these conditions [2–4]. Importantly, most patients are still resistant or relapse to currently available pharmacological and/or cognitive behavioural therapies [5, 6].

Pavlovian fear conditioning is a translationally relevant model, key in both understanding the physiology of fear and the pathophysiology of anxiety disorders. Fear conditioning is an associative learning process in which a neutral stimulus, such as a tone or a specific context (conditioned stimulus, CS), is presented together with an aversive stimulus, usually a foot shock (unconditioned stimulus, US), to generate a conditioned fear response [7]. The fear acquisition phase is followed by a consolidation phase, lasting hours to days, during which memories are formed [8]. Subsequent exposure to the CS, in absence of the US, is sufficient to evoke freezing behaviour, demonstrating the effective formation of a conditioned fear memory. Re-exposure to the CS after the stabilization of fear memories causes them to become labile and susceptible to disruption [9]. In this state, depending on the characteristics of the CS exposure, memories can progress to extinction, or they can undergo reconsolidation. Reconsolidation is the process through which memories re-stabilize after having been destabilized by exposure to the CS. It is thought to allow the integration of new information into the memory trace, and shares most, but not all, molecular mechanisms with fear consolidation [10, 11].

Fear conditioning is mediated by the brain fear circuit, a complex ensemble of brain areas with highly specialized cell populations and an intricate connectivity [12, 13]. The core brain region of the fear circuit is the amygdala, that is implicated in fear conditioning in animals [14, 15] and humans [16, 17]. Other fundamental regions of the fear circuit are the medial prefrontal cortex (mPFC), involved in fear retrieval and extinction [12, 13], and the hippocampus that encodes information about the context [18, 19].

Fear memory acquisition and consolidation are achieved through an assortment of cellular and molecular mechanisms, many of them underlying synaptic plasticity at fear network brain regions [20]. Of note, the MAPK/Erk pathway and the PI3-K/Akt pathway are essential for the consolidation of fear memories [21–23], by regulating transcription of genes that promote synaptic plasticity [24].

The MAPK/Erk pathway, the PI3-K/Akt pathway and the PLC-γ/Ca^2+^ pathway [25, 26] are the three main intracellular signalling cascades initiated by neurotrophins. Neurotrophins are a family of growth factors classically known for their role in neuronal development [27] and their potential to promote synaptic plasticity [28, 29]. Mature neurotrophins, i.e. nerve growth factor (NGF), brain-derived neurotrophic factor (BDNF), neurotrophin-3 (NT-3) and neurotrophin-4/5 (NT-4/5), selectively bind receptors of the tropomyosin receptor kinase (Trk) family with high affinity [29, 30]. TrkA preferentially binds NGF, TrkB preferentially binds BDNF and NT-4, while TrkC preferentially binds NT-3 [26, 29, 31].

The activation of the downstream pathways mentioned above regulates gene expression and promotes synaptic plasticity, placing neurotrophins as good candidates in the regulation of fear memory. In fact, BDNF and its receptor TrkB have been shown to be necessary for proper fear learning [32–35]. The NT-3/TrkC system is less consistently studied, but several lines of evidence from our laboratory and others strongly suggest that the NT-3/TrkC pathway is also involved in the regulation of fear in anxiety and fear-related disorders. First, human genetic studies found an association of polymorphisms in *NTRK3*, the gene that codes for TrkC, with panic disorder patients [36, 37]. Studies in mice have further supported a role for NT-3/TrkC in panic disorder. A transgenic mouse (Tg*NTRK3*) that overexpresses human TrkC, validated as a panic disorder model, shows increased anxiety-related behaviours [38] and increased contextual fear memory that is resistant to extinction processes [39, 40]. More recently, a study in *Rhesus monkeys* showed that *NTRK3* expression levels in the dorsal amygdala are inversely correlated with anxious temperament, a risk factor for the development of anxiety disorders [41]. Notably, NT-3 overexpression in the amygdala rescues anxious temperament levels [41].

Here, we aimed at investigating the NT-3/TrkC system in the formation of a contextual fear memory. C57Bl/6 J mice were trained in the contextual fear conditioning paradigm to study the NT-3/TrkC system during the time windows of consolidation and reconsolidation of a fear memory in the amygdala-hippocampus-PFC fear network.

## Material & Methods

### Animals

A total of 57 C57Bl/6J male mice (8 weeks of age) were used in this study (purchased from Charles River laboratories). Mice were housed in groups of four (eventually two) animals per cage containing sawdust, paper bag and cardboard roll as nesting material and shelter. Food and water were available ad libitum and animals were maintained in a 12-h light/dark cycle, with controlled conditions of temperature (18-22ºC) and humidity (60–70%). All described procedures were carried out in strict accordance with the EU directive 2010/63/EU and approved by the local ethical committee for animal well-being and experimentation (ORBEA, project number 209/2018).

### Contextual Fear Conditioning Paradigm

Animals were trained in the contextual fear conditioning (CFC) paradigm as previously described [39, 40]. Mice were transferred to the behaviour room three days before the beginning of the behavioural test for acclimation. On day one, mice were placed in the fear conditioning chamber (UgoBasile, Italy) for 3 min for habituation. On day 2, CFC training consisted in 2 min of exploration of the chamber, during which basal freezing levels were recorded, followed by administration of 5 foot-shocks (US1-US5, 0.5 mA, 2 s), separated by a variable inter-trial interval (between 15–60 s). Freezing was manually scored in the 15 s following each shock. Twenty-four hours after CFC training, fear conditioned and control mice were placed back in the chamber for 2 min, during which freezing was scored to assess fear memory retrieval. The chamber was cleaned with 10% ethanol to provide a neutral olfactory environment. Animals in the control groups were treated the same way as animals in the experimental groups, but did not receive any shocks (CTRL-no shock group).

### Sample Collection and Processing

Animals in the experimental groups and their respective controls were sacrificed 2–4 h after CFC training (fear acquisition group) or after fear memory retrieval (fear memory group), and the hippocampi, PFC and amygdalae were isolated and immediately frozen in dry ice and stored at -80 °C until further processing. In particular, PFC dissection includes approximately 2.5 mm of the most frontal part of the brain (coordinates from Paxinos & Franklin mouse brain atlas [42]: anterior–posterior + 3.3 to + 1.8), excluding the portion of the olfactory bulbs, olfactory areas and nuclei.

For total protein extraction, brain tissues were mechanically homogenized in radioimmunoprecipitation assay (RIPA) buffer (150 mM NaCl, 50 mM Tris–HCl pH7.4, 5 mM EGTA, 1% Triton, 0.5% DOC and 0.1% SDS, pH7.5) supplemented with protease (Complete protease inhibitor cocktail, Roche, Switzerland) and phosphatase (PhosSTOP, Sigma-Aldrich, MA, USA) inhibitors. Lysates were left at an orbital rotator for 30 min at 4 °C and then centrifuged at 16,000 × g for 30 min at 4 °C. The supernatant was collected and stored at -80 °C. Total protein content was quantified using the bicinchoninic acid (BCA) protein assay kit (Sigma-Aldrich, MA, USA).

### Western Blot

Total protein extracts (30 μg) were resolved in 7% sodium dodecyl sulfate–polyacrylamide gels and transferred to polyvinylidene fluoride (PVDF) membranes (Immobilon-P, Merck Millipore, MA, USA) overnight at 40 V, plus 30 min at 100 V, at 4 °C. Membranes were then blocked for 1 h at room temperature (RT) in 5% (w/v) low fat milk prepared in tris-buffered saline-tween-20 (TBS-T: 137 mM NaCl, 20 mM tris–HCl, pH 7.6 with 0.1% tween-20), followed by overnight incubation at 4 °C with primary antibodies diluted in 5% milk/TBS-T. Membranes were washed with TBS-T, and incubated for 90 min at RT with appropriate secondary antibodies diluted in 0.5% milk/TBS-T. After washing, membranes were incubated with ECF substrate (GE Healthcare, IL, USA) and scanned with the ChemiDoc imaging system (Bio-Rad, CA, USA). Membranes were stripped of primary and secondary antibodies using 0.2 M NaOH (20 min at RT), blocked 1 h at RT in 5% milk/TBS-T, and probed for additional proteins of interest. β-actin detection was used as a loading control. Bands were quantified using ImageJ software (National Institutes of Health, MD, USA) following the guidelines of Gassmann and colleagues [43]. The following primary antibodies were used in this study: rabbit anti-phospho-TrkC Tyr516 (PA5-39755, 1:500 dilution, Thermo Fisher Scientific, MA, USA), rabbit anti-TrkC (3376, 1:1000, Cell Signaling Technology, MA, USA), mouse anti-diphosphorylated-MAPK (M9692, 1:3000, Sigma-Aldrich, MA, USA), rabbit anti-MAPK (M5670, 1:20,000, Sigma-Aldrich, MA, USA), rabbit anti-phospho-Akt Ser473 (9271, 1:2000, Cell Signaling Technology, MA, USA), rabbit anti-Akt (sc-8312, 1:500, Santa Cruz Biotechnology, TX, USA), mouse anti-PLC-γ (610027, 1:1000, BD Transduction Laboratories, NJ, USA), mouse anti-PTP1B (sc-133259, 1:1000, Santa Cruz Biotechnology, TX, USA) and mouse anti-β-actin antibody (A5441, 1:5000, Sigma-Aldrich, MA, USA). The following secondary antibodies were used in this study: alkaline phosphatase-conjugated antibody anti-rabbit (A16026, 1:10,000, Thermo Fisher Scientific, MA, USA) or anti-mouse (A16014, 1:10,000, Thermo Fisher Scientific, MA, USA).

### Enzyme-Linked Immunosorbent Assay (ELISA)

A NT-3 ELISA kit (#BEK-2079-2P, Biosensis, Australia) was used to assess the concentration of NT-3 in brain tissue lysates. Total protein extracts were diluted 1:5 in sample diluent buffer, plated in a 96-well microplate pre-coated with a monoclonal NT-3 antibody and incubated overnight at 4 °C. After 5 washes with phosphate-buffered saline (PBS), a 2 h incubation with biotinylated anti-NT-3 antibody was performed. The wells were washed 3 with PBS and incubated with avidin–biotin-peroxidase complex (ABC) enzyme for 1 h at RT. After 5 washes with PBS, the peroxidase substrate TMB was added. The reaction was stopped after 10 min by adding TBM stop solution. Absorbance was measured at 450 nm using a microplate spectrophotometer (SpectraMax Plus 384, Molecular Devices, CA, USA).

### Statistical Analysis

All data were analysed using GraphPad Prism 8 software (Version 8.4.3, GraphPad Software, CA, USA). The normality of each data set was assessed using the Shapiro–Wilk test and outliers (defined by the ROUT method, Q = 1%) were removed from normal distributions. CFC data were analysed using repeated measures two-way ANOVA with post hoc Bonferroni’s test for pairwise comparisons (fear acquisition groups) and Student’s *t*-test (fear memory groups). For western blot and ELISA data, the means (in the case of normal distribution) or the ranks (in the case of non-normal distribution) of each condition were compared using Student’s *t*-test or Mann–Whitney U test, respectively. For Student’s *t*-test, Welch correction was applied in the case of significant difference of variances. For each western blot, data were normalized to the mean of CTRL-no shock group. Graphs represent average values ± standard error of the mean (SEM). Statistical significance was set at 0.05 (*p ≤ 0.05, **p ≤ 0.01, ***p ≤ 0.001).

## Results

C57BI/6J mice were trained in the CFC paradigm. The fear acquisition group and respective control animals (fear acquisition, *n* = 18; CTRL-no shock, *n* = 8) were sacrificed 2 to 4 h after fear conditioning, in the window of fear memory consolidation. The fear memory group and respective controls (fear memory, *n* = 21; CTRL-no shock, *n* = 10) performed a fear memory retrieval test 24 h after fear training, to assess the retention of fear memory. Here, animals were sacrificed 2 to 4 h after fear memory retrieval, in the window of reconsolidation. Sacrifice of control and test animals was randomized to avoid bias in sample collection. Our goal was to study alterations in NT-3/TrkC and its intracellular signalling during the formation of a contextual fear memory. To that end, we first quantified the levels of TrkC expression and activation (as measured by phosphorylation) in the brain fear circuit, comprising the amygdala, PFC and hippocampus, of fear conditioned mice during the periods of fear memory consolidation (fear acquisition group) and reconsolidation (fear memory group).

### Consolidation of a Fear Memory Correlates with Decreased TrkC Activation in the Amygdala and PFC

**Fig. 1:**
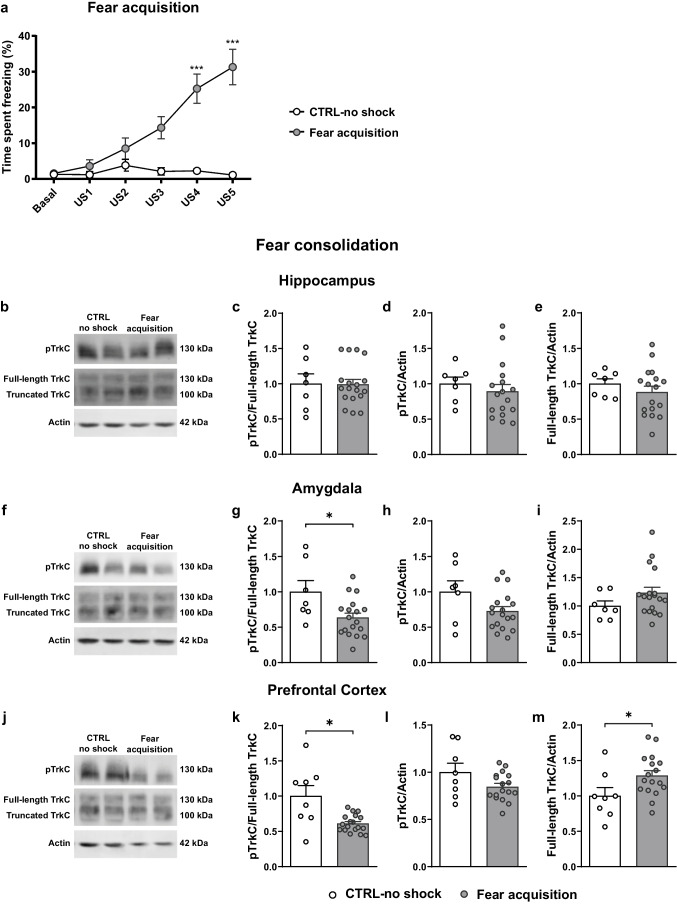
Expression and activation of TrkC in the fear circuit during contextual fear memory consolidation. **(a)** Fear acquisition mice (*n* = 18) underwent contextual fear conditioning, while CTRL-no shock mice (*n* = 8) did not receive any foot-shocks. The percentage of time spent freezing was assessed during the initial 2 min before US presentation (basal) and in the 15 s after each shock (US1-US5). **(b, f, j)** Representative western blot images of pTrkC and TrkC performed in **(b)** hippocampus, **(f)** amygdala and **(j)** prefrontal cortex total protein extracts from fear acquisition (*n* = 18) and CTRL-no shock (*n* = 8) mice sacrificed during fear consolidation. Quantification of **(c, g, k)** pTrkC/full-length TrkC ratio, **(d, h, l)** levels of phosphorylated TrkC and **(e, i, m)** total full-length TrkC levels. β-actin was used as a loading control. *p ≤ 0.05, **p ≤ 0.01, ***p ≤ 0.001. CTRL, control; pTrkC, phosphorylated TrkC; US, unconditioned stimulus

For the fear acquisition group (Fig. 1a), repeated measures two-way ANOVA performed on the freezing levels revealed a statistically significant effect of US presentations (US1-US5) (F _(2.974, 71.38)_ = 8.36, *p* < 0.0001), treatment (CTRL-no shock *vs* CFC, F _(1, 24)_ = 14.32, *p* = 0.0009) and US x treatment interaction (F _(5, 120)_ = 8.591, *p* < 0.0001). Post hoc comparisons revealed that freezing levels increased with successive shock presentations (CFC: US1 *vs* US5, t _(17)_ = 5.387, *p* = 0.0007) and that the percentage of time freezing in the fear acquisition animals was increased as compared to CTRL-no shock animals (US5: CTRL-no shock *vs* CFC, t _(17.43)_ = 6.049, *p* < 0.0001), confirming that a fear response was successfully induced in the animals in the experimental condition.

During fear memory consolidation, no significant differences were found in the expression and phosphorylation levels of TrkC between fear acquisition and CTRL-no shock mice in the hippocampus (pTrkC, t _(22)_ = 0.6664, *p* = 0.5121; full-length TrkC, t _(22)_ = 0.8627, *p* = 0.3976; pTrkC/full-length TrkC ratio, U = 59, *p* = 0.8362; Fig. 1b-e). However, in the amygdala (Fig. 1f-i) we observed a significant decrease in the relative phosphorylation of TrkC in fear acquisition animals when compared to CTRL-no shock (pTrkC/full-length TrkC ratio, t _(23)_ = 2.583, *p* = 0.0166) and a trend for decrease in the total levels of phosphorylated TrkC (t _(23)_ = 1.945, *p* = 0.0641), while the levels of full-length TrkC showed no alterations (U = 46, *p* = 0.3261). Likewise, in the PFC (Fig. 1j-m) contextual fear conditioning is associated with a decrease in the pTrkC/full-length TrkC ratio (t _(7.513)_ = 2.531, *p* = 0.037), accompanied by a significant increase in the levels of full-length TrkC (t _(23)_ = 2.184, *p* = 0.0394), but no differences in total phosphorylated TrkC (t _(23)_ = 1.850, *p* = 0.0772).

### Reconsolidation of a Fear Memory Correlates with Decreased TrkC Activation in the Hippocampus

**Fig. 2:**
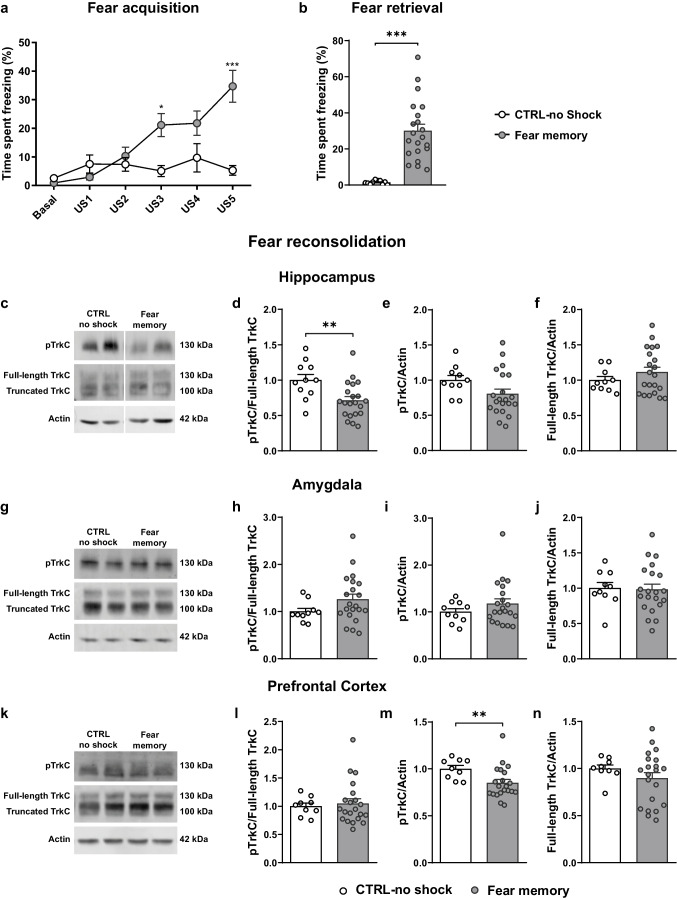
Expression and activation of TrkC in the fear circuit during contextual fear memory reconsolidation. **(a)** Fear memory mice (*n* = 21) underwent contextual fear conditioning, while CTRL-no shock mice (*n* = 10) did not receive any foot-shocks. The percentage of time spent freezing was assessed during the initial 2 min before US presentation (basal) and in the 15 s after each shock (US1-US5). **(b)** Percentage of time spent freezing was measured in conditioned and control mice during a 2-min fear retrieval session 24 h after CFC. **(c, g, k)** Representative western blot images of pTrkC and TrkC performed in **(c)** hippocampus, **(g)** amygdala and **(k)** prefrontal cortex total protein extracts from fear memory (*n* = 21) and CTRL-no shock (*n* = 10) mice sacrificed during fear reconsolidation. Quantification of **(d, h, l)** pTrkC/full-length TrkC ratio, **(e, i, m)** levels of phosphorylated TrkC and **(f, j, n)** total full-length TrkC levels. β-actin was used as a loading control. *p ≤ 0.05, **p ≤ 0.01, ***p ≤ 0.001. Panel **c**), non-contiguous lanes from the same membrane. CFC, contextual fear conditioning; CTRL, control; pTrkC, phosphorylated TrkC; US, unconditioned stimulus

In the fear memory group, during the fear acquisition phase (Fig. 2a), we observed a significant effect of US presentations (F _(5, 145)_ = 8.919, *p* < 0.0001), treatment (F _(1, 29)_ = 6.166, *p* = 0.0191) and US x treatment interaction (F _(5, 145)_ = 7.810, *p* < 0.0001) in the percentage of time spent freezing. Again, successive US presentations increased the time spent freezing in the fear conditioned animals (US1 *vs* US5, t _(145)_ = 8.684, *p* < 0.0001) and the percentage of time freezing in the fear memory animals was increased as compared to CTRL-no shock animals (US5: CTRL-no shock *vs* CFC, t _(174)_ = 5.318, *p* < 0.0001). Twenty-four hours later, animals were placed back into the training chamber to assess fear memory retrieval. Here, conditioned mice displayed significantly higher freezing levels than CTRL-no shock mice (Fig. 2b, t _(20.33)_ = 7.869, *p* < 0.0001), demonstrating proper retention and recall of fear memory.

During fear memory reconsolidation, we observed a decrease in relative TrkC phosphorylation levels in the hippocampus of conditioned animals (t _(30)_ = 3.018, *p* = 0.0051) and a trend for decrease in total levels of phosphorylated TrkC (t _(29)_ = 1.780, *p* = 0.0856), with no differences observed in the levels of full-length TrkC (t _(28.74)_ = 1.319, *p* = 0.1974; Fig. 2c-f). In the amygdala (Fig. 2g-j), overall no differences were detected in the expression or activation levels of TrkC (pTrkC/full-length TrkC ratio, t _(28.84)_ = 1.992, *p* = 0.0559; full-length TrkC, t _(29)_ = 0.1586, *p* = 0.8751; pTrkC, U = 89, *p* = 0.5189). In the PFC (Fig. 2k-n), the total levels of phosphorylated TrkC were significantly decreased in conditioned animals (U = 33, *p* = 0.0042), with no differences found in the relative phosphorylation (U = 88, *p* = 0.7899) or in the levels of full-length TrkC (t _(28)_ = 1.43, *p* = 0.1639).

Overall, our data points to a downregulation of TrkC signalling in key brain regions of the fear network during the formation of a fear memory.

### Fear Memory-Related Decrease in TrkC Phosphorylation in the Brain Fear Circuit is not Associated with a Reduction in NT-3 Levels or Increase in Truncated TrkC Isoform

**Fig. 3:**
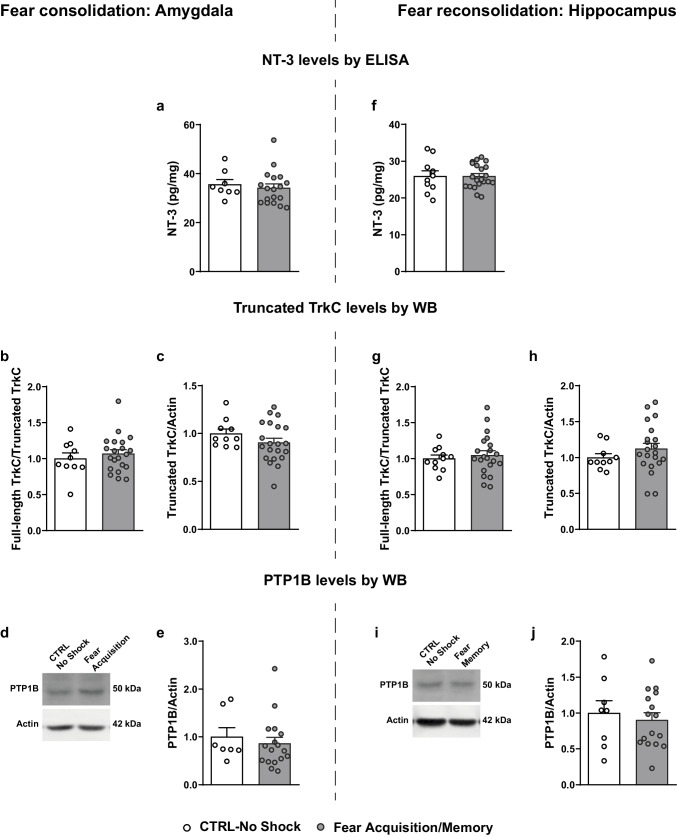
Expression levels of different modulators of TrkC activation. **(a, f)** Quantification by ELISA of NT-3 levels (pg/mL) in protein extracts from **(a)** the amygdala of fear acquisition animals sacrificed during fear consolidation and **(f)** the hippocampus of fear memory animals sacrificed during fear reconsolidation, and respective controls. **(b, c, g, h)** Quantification of **(b, g)** full-length TrkC/truncated TrkC ratio and **(c, h)** truncated TrkC levels in total protein extracts from **(b, c)** the amygdala of fear acquisition animals sacrificed during fear consolidation and **(g, h)** the hippocampus of fear memory animals sacrificed during fear reconsolidation. Representative western blot images showing full-length TrkC and truncated TrkC under CTRL-no shock and fear acquisition/fear memory conditions are shown in Figs. 1f and 2c. **(d, i)** Representative images of PTP1B western blot performed in total protein extracts from **(d)** the amygdala of fear acquisition animals sacrificed during fear consolidation and **(i)** the hippocampus of fear memory animals sacrificed during fear reconsolidation. **(e, j)** Quantification of expression levels of PTP1B. β-actin was used as a loading control in western blots. CTRL, control; ELISA, enzyme-linked immunosorbent assay; NT-3, neurotrophin 3; WB, western blot

Next, we used ELISA to measure the levels of NT-3, which binds TrkC with high affinity, in lysates from brain regions highly implicated in the processing of fear and where the relative activation of TrkC was found to be altered at specific timepoints, i.e. amygdala during the fear consolidation phase (Fig. 1f, g) and hippocampus during fear reconsolidation (Fig. 2c, d). Statistical analysis did not reveal differences in the NT-3 levels between fear acquisition and CTRL-no shock animals in the amygdala (U = 60, *p* = 0.5308; Fig. 3a) or between fear memory and CTRL-no shock animals in the hippocampus (t _(29)_ = 0.014, *p* = 0.9889; Fig. 3f). Although we did not observe differences in total NT-3 levels, both during consolidation and reconsolidation of fear, we cannot exclude changes in the activity-dependent secretion of NT-3 that are more likely to account for synaptic plasticity effects.

The truncated isoform of TrkC, which cannot be phosphorylated, can act as a dominant negative by sequestering NT-3 and preventing the activation of full-length TrkC receptors [44, 45]. To investigate the possibility that the overall decrease in TrkC phosphorylation, observed in the brain regions of the fear circuit of fear conditioned animals, is accompanied by an increase of the dominant negative truncated TrkC isoform, we measured its expression levels. During fear consolidation, no differences were detected between fear acquisition and CTRL-no shock animals in the ratio of full-length TrkC/truncated TrkC (U = 60, *p* = 0.8826) or in the expression levels of truncated TrkC (t _(23)_ = 1.059, *p* = 0.3007) in the amygdala (Figs. 1f and 3b, c). Likewise, no differences were found between groups in the hippocampus (full-length TrkC/truncated TrkC, t _(23)_ = 0.0354, *p* = 0.9721; truncated TrkC, t _(23)_ = 0.2729, *p* = 0.7874; Fig. 1c; supplementary Fig. 1a, b) or in the PFC (full-length TrkC/truncated TrkC, U = 46, *p* = 0.1597; truncated TrkC, t _(24)_ = 1.349, *p* = 0.19; Fig. 1j; supplementary Fig. 1c, d).

During fear reconsolidation, also no differences were observed in the total or relative expression levels of truncated TrkC between fear memory and CTRL-no shock animals in the hippocampus (full-length TrkC/truncated TrkC, t _(30)_ = 0.5184, *p* = 0.6080; truncated TrkC, t _(28.88)_ = 1.319, *p* = 0.1974; Figs. 2c and 3g, h), amygdala (full-length TrkC/truncated TrkC, t _(29)_ = 0.6821, *p* = 0.5006; truncated TrkC, t _(29)_ = 1.270, *p* = 0.2143; Fig. 2g; supplementary Fig. 1e, f) or PFC (full-length TrkC/truncated TrkC, U = 87, *p* = 0.7558; truncated TrkC, t _(27.21)_ = 0.8677, *p* = 0.3932; Fig. 2k; supplementary Fig. 1 g, h).

Overall, there is no evidence for an association between the observed decrease in TrkC phosphorylation levels, during the consolidation and reconsolidation of a contextual fear memory, and alterations in the expression of TrkC truncated isoform.

### No Alterations in the Expression Levels of the Trk-Targeting Phosphatase PTP1B

PTP1B is a phosphatase that targets, among others, Trk receptors, dephosphorylating them [46]. To investigate the possibility that the observed decrease in TrkC activation during the (re)consolidation of a contextual fear memory is associated with an increased expression of the TrkC-targeting PTP1B phosphatase, after fear acquisition or fear memory retrieval, we measured its expression levels, by western blot, in brain regions and timepoints where a decrease in TrkC activation was observed. We did not observe any differences between conditioned and CTRL-no shock animals in the levels of PTP1B in the amygdala during fear consolidation (U = 47, *p* = 0.4551; Fig. 3d, e) or in the hippocampus during fear reconsolidation (t _(22)_ = 0.5175, *p* = 0.61; Fig. 3i, j). These results provide no evidence for an association between the observed decrease in TrkC phosphorylation levels, during the consolidation and reconsolidation of a contextual fear memory, and alterations in the expression of PTP1B.

### Downregulation of Hippocampal NT-3/TrkC-ERK Pathway During Reconsolidation of Contextual Fear Memory

Given the results described above showing alterations in TrkC activation associated with the formation of a contextual fear memory, we aimed at investigating possible alterations in intracellular signalling pathways activated by the NT-3/TrkC system. To this end, we measured the expression and phosphorylation levels of the endpoint molecules Erk, Akt and PLC-γ in brain regions of the fear network during fear consolidation and fear reconsolidation timepoints.

**Fig. 4:**
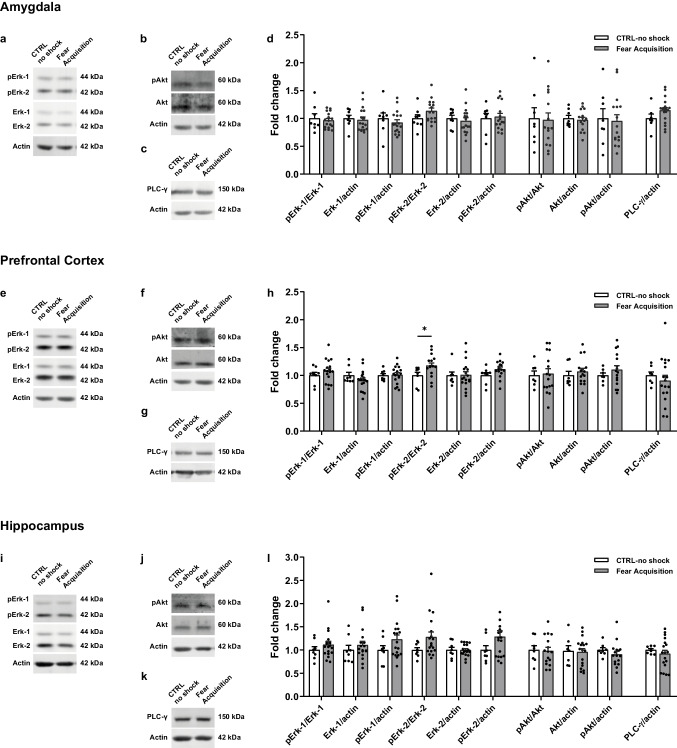
Expression levels of endpoint molecules of TrkC-recruited signalling pathways in the brain fear network during contextual fear consolidation. Representative western blot images of **(a, e, i)** pErk-1/2 and Erk-1/2, **(b, f, j)** pAkt and Akt, and **(c, g, k)** PLC-γ performed in the **(a-c)** amygdala, **(e–g)** prefrontal cortex and **(i-k)** hippocampus total protein extracts from fear acquisition (*n* = 18) and CTRL-no shock (*n* = 8) mice, sacrificed during fear consolidation. **(d, h, l)** Quantification of the ratio of phosphorylated/total protein levels, levels of phosphorylated proteins and total protein levels in **(d)** amygdala, **(h)** prefrontal cortex and **(l)** hippocampus. β-actin was used as a loading control. *p ≤ 0.05. CTRL, control

During the consolidation phase, in the amygdala no differences were found between fear acquisition and CTRL-no shock animals in the expression and phosphorylation of Erk (Fig. 4a, d; pErk-1/Erk-1, t _(23)_ = 0.4727, *p* = 0.6409; total Erk-1, U = 55, *p* = 0.4747; pERK-1, t _(23)_ = 0.7439, *p* = 0.4645; pErk-2/Erk-2, U = 40, *p* = 0.1104; total Erk-2, t _(23)_ = 0.4921, *p* = 0.6273; pErk-2, U = 60, *p* = 0.834), Akt (Fig. 4b, d; pAkt/Akt, t _(23)_ = 0.1627, *p* = 0.8722; total Akt, t _(22)_ = 0.2265, *p* = 0.8229; pAkt, U = 65, *p* = 0.8867), or in the expression of total PLC-γ (Fig. 4c, d; t _(23)_ = 1.5, *p* = 0.1473).

In the PFC, there were no differences between fear acquisition and CTRL-no shock animals in the expression and phosphorylation of Erk-1 (Fig. 4e, h; pErk-1/Erk-1, t _(23)_ = 0.9776, *p* = 0.3385; total Erk-1, U = 57, *p* = 0.4285; pErk-1, t _(24)_ = 0.1666, *p* = 0.8691). We did observe an increase in the pErk-2/Erk-2 ratio in fear acquisition animals as compared to CTRL-no shock (Fig. 4e, h; U = 24, *p* = 0.0131), though we did not detect differences between groups in the levels of total Erk-2 or phosphorylated Erk-2 (Fig. 4e, h; total Erk-2, t _(24)_ = 0.0991, *p* = 0.9219; pErk-2, t _(22)_ = 1.739, *p* = 0.096). No differences between groups were observed in the expression and phosphorylation of Akt (Fig. 4f, h; pAkt/Akt, t _(21)_ = 0.2019, *p* = 0.842; total Akt, U = 41, *p* = 0.3411; pAkt, t _(21)_ = 1.18, *p* = 0.2512) or in the expression of total PLC- γ (Fig. 4g, h; t _(22.94)_ = 0.8122, *p* = 0.425).

In the hippocampus, we did not observe any differences between fear acquisition and CTRL-no shock experimental groups in the expression and phosphorylation of Erk-1/2 (Fig. 4i, l; pErk-1/Erk-1, U = 55, *p* = 0.367; total Erk-1, U = 55, *p* = 0.367; pErk-1, t _(24)_ = 1.416, *p* = 0.1696; pErk-2/Erk-2, U = 42, *p* = 0.1021; total Erk-2, t _(24)_ = 0.0333, *p* = 0.9737; pErk-2, U = 52, *p* = 0.2852), Akt (Fig. 4j, l; pAkt/Akt, t _(22)_ = 0.1373, *p* = 0.8921; total Akt, t _(23)_ = 0.1541, *p* = 0.8789; pAkt, t _(22)_ = 0.9498, *p* = 0.3525) or in the expression of total PLC- γ (Fig. 4k, l; t _(20.96)_ = 0.9924, *p* = 0.3323).

In sum, during the consolidation phase we observed an increase in the pErk2/Erk2 ratio in the PFC of fear acquisition animals as compared to CTRL-no shock animals, where TrkC changes were not detected, indicating that two pathways are most probably unrelated in these conditions.

**Fig. 5:**
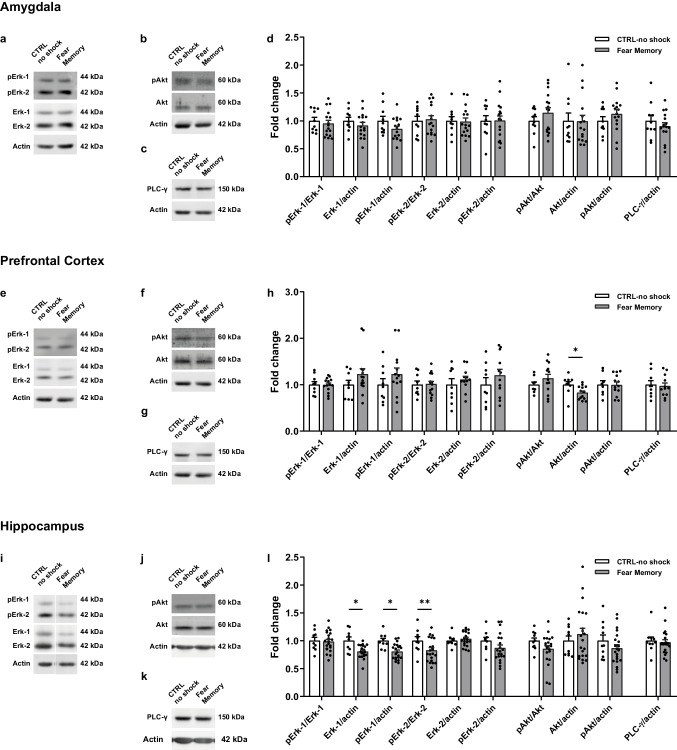
Expression levels of endpoint molecules of TrkC-recruited signalling pathways in the brain fear network during contextual fear reconsolidation. Representative western blot images of **(a, e, i)** pErk-1/2 and Erk-1/2, **(b, f, j)** pAkt and Akt and **(c, g, k)** PLC-γ performed in the **(a-c)** amygdala, **(e–g)** prefrontal cortex and **(i-k)** hippocampus total protein extracts from fear memory (*n* = 16–21) and respective CTRL-no shock (*n* = 10) mice, sacrificed during fear reconsolidation. **(d, h, l)** Quantification of the ratio of phosphorylated/total protein levels, levels of phosphorylated proteins and total protein levels in the **(d)** amygdala, **(h)** prefrontal cortex and **(l)** hippocampus. β-actin was used as a loading control. *p ≤ 0.05, **p ≤ 0.01. CTRL, control; panel i), non-contiguous lanes from the same membrane

During the reconsolidation window, in the amygdala we did not detect differences between fear memory animals and CTRL-no shock in any of the molecules studied. In detail, there were no differences between groups in the expression and phosphorylation levels of Erk-1/2 (Fig. 5a, d; pErk-1/Erk-1, t _(24)_ = 0.5526, *p* = 0.5856; total Erk-1, t _(24)_ = 0.984, *p* = 0.3349; pErk-1, t _(24)_ = 1.649, *p* = 0.1121; pErk-2/Erk-2, t _(24)_ = 0.2231, *p* = 0.8254; total Erk-2, t _(24)_ = 0.1558, *p* = 0.8775; pErk-2, t _(24)_ = 0.0318, *p* = 0.9749), Akt (Fig. 5b, d; pAkt/Akt, t _(24)_ = 1.129, *p* = 0.27; total Akt, U = 77, *p* = 0.8971; pAkt, t _(24)_ = 1.081, *p* = 0.2904) or in the expression of total PLC- γ (Fig. 5c, d; t _(24)_ = 0.8548, *p* = 0.4011).

In the PFC, no differences were found in the expression and phosphorylation of Erk-1/2 between fear memory and CTRL-no shock groups (Fig. 5e, h; pErk-1/Erk-1, t _(21)_ = 0.0792, *p* = 0.9376; total Erk-1, U = 48, *p* = 0.3686; pErk-1, t _(21)_ = 1.145, *p* = 0.2652; pErk-2/Erk-2, t _(21)_ = 0.1265, *p* = 0.9005; total Erk-2, t _(19)_ = 0.816, *p* = 0.4246; pErk-2, t _(19)_ = 1.002, *p* = 0.3292). We observed a decrease in total Akt expression in the fear memory group (Fig. 5f, h; total Akt, t _(20)_ = 2.311, *p* = 0.0316), without differences in the phosphorylation of Akt (Fig. 5f, h; pAkt/Akt, t _(19)_ = 1.085, *p* = 0.2916; pAkt, t _(19)_ = 0.0506, *p* = 0.9601). We did not observe differences in the expression levels of total PLC- γ (Fig. 5g, h; t _(19)_ = 0.273, *p* = 0.7878).

In the hippocampus, we observed a significant decrease in the expression levels of total Erk-1 (Fig. 5i, l; t _(28)_ = 3.368, *p* = 0.0022) and in the levels of phosphorylated Erk-1 (t _(28)_ = 3.545, *p* = 0.0014) in fear memory animals, although no differences were found in the pErk-1/Erk-1 ratio (t _(28)_ = 0.1893, *p* = 0.8512). In addition, we observed a decrease in the pErk-2/Erk-2 ratio in fear memory animals (t _(28)_ = 2.062, *p* = 0.0486), without differences observed in the expression levels of total Erk-2 (t _(28)_ = 0.7121, *p* = 0.4823) or levels of phosphorylated Erk-2 (t _(28)_ = 1.424, *p* = 0.1655) between the two conditions. Furthermore, no differences were found in the expression or phosphorylation levels of Akt (Fig. 5j, l; pAkt/Akt, t _(29)_ = 1.569, *p* = 0.1274; total Akt, U = 102, *p* = 0.9173; pAkt, t _(29)_ = 1.16, *p* = 0.2555) or in the expression levels of total PLC-γ (Fig. 5k, l; U = 85, *p* = 0.4164).

In sum, we observed an overall decrease in the activation of Erk-1/2 in the hippocampus during fear reconsolidation concurrent with a decrease in the activation of TrkC. Also, the observed reduction in the expression of Akt in the PFC should not be related to TrkC signalling as no changes in TrkC activation were detected in the PFC and this pathway is not expected to affect Akt expression levels.

## Discussion

The fear conditioning paradigm has been a powerful model to study the neuronal mechanisms of fear learning and memory. A distributed network of brain regions is involved in learning and expressing fear responses (reviewed in [13]). In the present work, we focus on the amygdala, PFC and hippocampus, brain regions recruited during learned fear and sites of intense synaptic plasticity events, where neurotrophins play a critical role. We report an overall downregulation of TrkC activation in the brain fear network both in the consolidation and reconsolidation phases of fear memory formation, as compared to their respective controls. Moreover, we did not find differences in the levels of NT-3, the dominant negative truncated TrkC isoform or the TrkC-targeting phosphatase PTP1B, between fear conditioned and control animals, factors that could explain the observed differences in TrkC activation. Though we cannot completely exclude their involvement as we did not assess activity-dependent NT-3 release, truncated/full-length TrkC interaction or PTP1B activity. Importantly, during reconsolidation hippocampal TrkC downregulation was accompanied by a downregulation of ERK, suggesting a potential role for this intracellular signalling pathway in the physiological regulation of fear reconsolidation, which deserves further investigation.

### Decreased Activation of TrkC in the Fear Circuit During the (re)Consolidation of a Fear Memory

As mentioned above, we found here an overall decrease in TrkC activation in brain regions of the fear circuit, during the formation of a contextual fear memory. In particular, the most relevant differences were observed in the amygdala during the consolidation phase and in the hippocampus during the reconsolidation phase of fear memory. In fact, consolidation and reconsolidation of contextual fear memories are dually dissociable processes that recruit different pathways. While BDNF is selectively required for consolidation, the transcription factor Zif268 is selectively required for reconsolidation [10]. Contextual fear retrieval induces expression of several activity regulated genes (Zif268, c-Fos, and JunB) specifically in the CA1 dorsal hippocampus [47, 48], which are thought to regulate neuronal plasticity during reconsolidation. The observed hippocampal alterations in TrkC activation during contextual fear memory reconsolidation are consistent with a specific role of this region in the process of reconsolidation, mediated by regulation of neuronal plasticity. Consolidation of contextual fear memories, on the other hand, occurs both in the basolateral amygdala and the hippocampus. However, it is thought that initial consolidation occurs in the amygdala, and only afterwards the representation of the memory is projected to the hippocampus, consolidating for a second time here, a process essential for retention of long-term memories [49]. In fact, regulation of synaptic plasticity in the amygdala after contextual fear conditioning is key to the consolidation and long-term retention of fear memories [50], consistent with our results showing alterations in TrkC in the amygdala 2–4 h after contextual fear conditioning. In future work it would be important to show a causal link between TrkC inactivation and fear (re)consolidation.

### Decrease in TrkC Activation is Accompanied by Decreased Erk Expression and Phosphorylation in the Hippocampus

TrkC activation recruits three main intracellular signalling pathways whose endpoint molecules are Erk-1/2, Akt and PLC-γ [26]. We studied the levels of expression (for Erk-1/2, Akt and PLC-γ) and activation (for Erk-1/2 and Akt) of these proteins in brain regions where we have observed alterations in the activation of TrkC. During fear consolidation, we did not observe any differences in Erk1/2 expression or activation in the amygdala between fear conditioning and control animals. During fear reconsolidation, we observed an overall decrease in the activation of Erk-1/2, which is concurrent with a decrease in TrkC activation, suggesting that they might be correlated. A previous study found that there is a biphasic hippocampal activation of Erk critical in the consolidation of contextual fear memory [51], with peaks 15 min and 9 h after training. In between, 3 to 6 h after training, the pErk/Erk ratio is back to basal levels. Although consolidation and reconsolidation are two different processes, mediated by distinct mechanisms, we could speculate that TrkC could contribute to a necessary phase of downregulation of Erk phosphorylation during fear memory reconsolidation.

Overall, we did not observe differences in the expression and phosphorylation of Akt. Previously, an increased activation of Akt was observed in the amygdala 15 min after contextual fear conditioning [22], however, to the best of our knowledge, the activation of Akt had never been studied at the later timepoint in focus in our study. Few studies have explored the role of PLC-γ in fear conditioning. One study found that PLC-γ activation is increased in the hippocampus 30 min after contextual fear conditioning [52]. Additionally, transgenic mice overexpressing TrkB show enhanced contextual fear learning, which is associated with an increase in the activation of PLC-γ1 in the hippocampus and the cerebral cortex [53]. Again, no study focused on the timepoint investigated. At this timepoint, we found no alterations in the expression of PLC-γ. However, we could not find a working antibody to study PLC-γ phosphorylation.

### Decrease in TrkC Activation is not Accompanied by Alterations in NT-3, Truncated TrkC or Phosphatase PTP1B Expression

Truncated Trks are known to have a dominant negative effect and thereby preclude full-length Trk signalling by sequestering neurotrophin ligands [45]. In this scenario, an increase in truncated TrkC expression could explain the observed reduction in TrkC activation, though we did not find differences in the expression levels of truncated TrkC between fear conditioned and control animals. However, a contribution of this isoform to the decreased TrkC activation observed during fear memory (re)consolidation cannot be excluded since the interaction between truncated and full-length TrkC was not directly assessed.

Another possible cause for the observed decrease in TrkC activation could lie in alterations in the expression or availability of its ligand, NT-3. We did not find alterations in NT-3 expression in the amygdala during fear consolidation and the hippocampus during fear reconsolidation, our two main focuses. In spite of this, we cannot exclude a role for NT-3 in the activation of TrkC during the formation of a fear memory. First, we measured NT-3 concentrations during the consolidation and reconsolidation phases, 2 to 4 h after fear training and fear retrieval, respectively, though the action of NT-3 may be more immediate, after which signalling is carried on by intracellular mechanisms. Second, we studied whole tissue protein extracts, while we know that brain regions of the fear circuit have highly specialized sub-circuits [12, 13]. It is possible that NT-3 may act locally and the alterations in its local availability may be diluted when we study the entire brain region. Of note, NT-3 is highly expressed in the hippocampus CA2 region [54], and is not expressed in the adult amygdala, unlike TrkC [55], suggesting that NT-3 travels from other brain regions to the amygdala to activate TrkC. In future studies it would be interesting to measure local secretion of NT-3 during performance in fear conditioning.

It is known that fear conditioning increases BDNF in the fear circuit, accompanied by activation of TrkB and recruitment of intracellular signalling pathways that will regulate synaptic plasticity. Briefly, contextual fear conditioning increases *BDNF* transcription in the CA1 region of the rat hippocampus [56, 57], while cued fear conditioning increases BDNF expression in the rat amygdala [58, 59]. Additionally, *Bdnf* heterozygous null (*Bdnf*^+/-^) mice display impaired contextual fear conditioning, which is partially rescued by chronic administration of recombinant BDNF into the hippocampus [35], and blocking activation of TrkB in the rat amygdala impairs fear memory consolidation [59] and retention [60]. Our data suggests that a possible role of NT-3/TrkC system in fear conditioning may differ from that of BDNF/TrkB.

Finally, TrkC inactivation is also not associated with an increase in the expression of the Trk-targeting phosphatase PTP1B [46], as we observed no differences in its levels. However, it is important to note that we did not measure the enzymatic activity of this phosphatase, which along with its level of expression is a main indicator of its impact on other proteins in the system. Other mechanisms could explain the decreased activation of TrkC, such as desensitization of TrkC receptors by internalization [61] and downregulation of TrkC cell-surface expression [62] that could be explored in the future.

In summary, TrkC activation is generally decreased in the brain fear circuit during fear consolidation and reconsolidation, while no alterations are observed in the expression of NT-3, truncated TrkC or PTP1B phosphatase. Furthermore, during reconsolidation, this decrease is accompanied by an overall decrease in Erk expression and activation in the hippocampus. This spatio-temporal characterization of TrkC activation state, and related up- and downstream factors, during fear (re)consolidation provides the first evidence associating TrkC with learned fear and per se represents a relevant contribution to the neurotrophin field, where for so many years the NT-3/TrkC system was left orphan. In the future, proving causality and elucidating the underlying molecular mechanisms will have a major impact on the field of fear by bringing into scene a new molecular player with potential in mental health promotion.

## Supplementary Information

Below is the link to the electronic supplementary material.

Supplementary file1 (DOCX 157 kb)

## Data Availability

All data generated or analysed during this study are included in this published article.
