# Supplementary material for: TrkC Intracellular Signalling in the Brain Fear Network During the Formation of a Contextual Fear Memory
Source: Mol Neurobiol. 2023 Mar 8;60(6):3507–21. doi: 10.1007/s12035-023-03292-0 (PMC10122637; doi:10.1007/s12035-023-03292-0)
Supplement: Supplementary file 1 — Supplementary file1 (DOCX 157 kb) [file 12035_2023_3292_MOESM1_ESM.docx]

TrkC intracellular signalling in the brain fear network during the formation of a contextual fear memory

Molecular Neurobiology

Francisca Silva^1, 2^, Gianluca Masella^1, 2^, Maria Francisca Madeira^1^, Carlos B. Duarte^1, 3^, Mónica Santos^1, 2, *^

1. CNC – Center for Neuroscience and Cell Biology, University of Coimbra, Coimbra, Portugal

2. Institute of Interdisciplinary Research, University of Coimbra (iiiUC), Coimbra, Portugal

3. Department of Life Sciences, University of Coimbra, Portugal

* Corresponding author

Mónica Santos

Email: [mjpsantos@cnc.uc.pt](mailto:mjpsantos@cnc.uc.pt); monicapsantos@hotmail.com

ORCID: 0000-0003-3229-8270

Center for Neuroscience and Cell Biology

Faculdade de Medicina, 2º piso

Universidade de Coimbra

Rua Larga

3004-504 Coimbra, Portugal


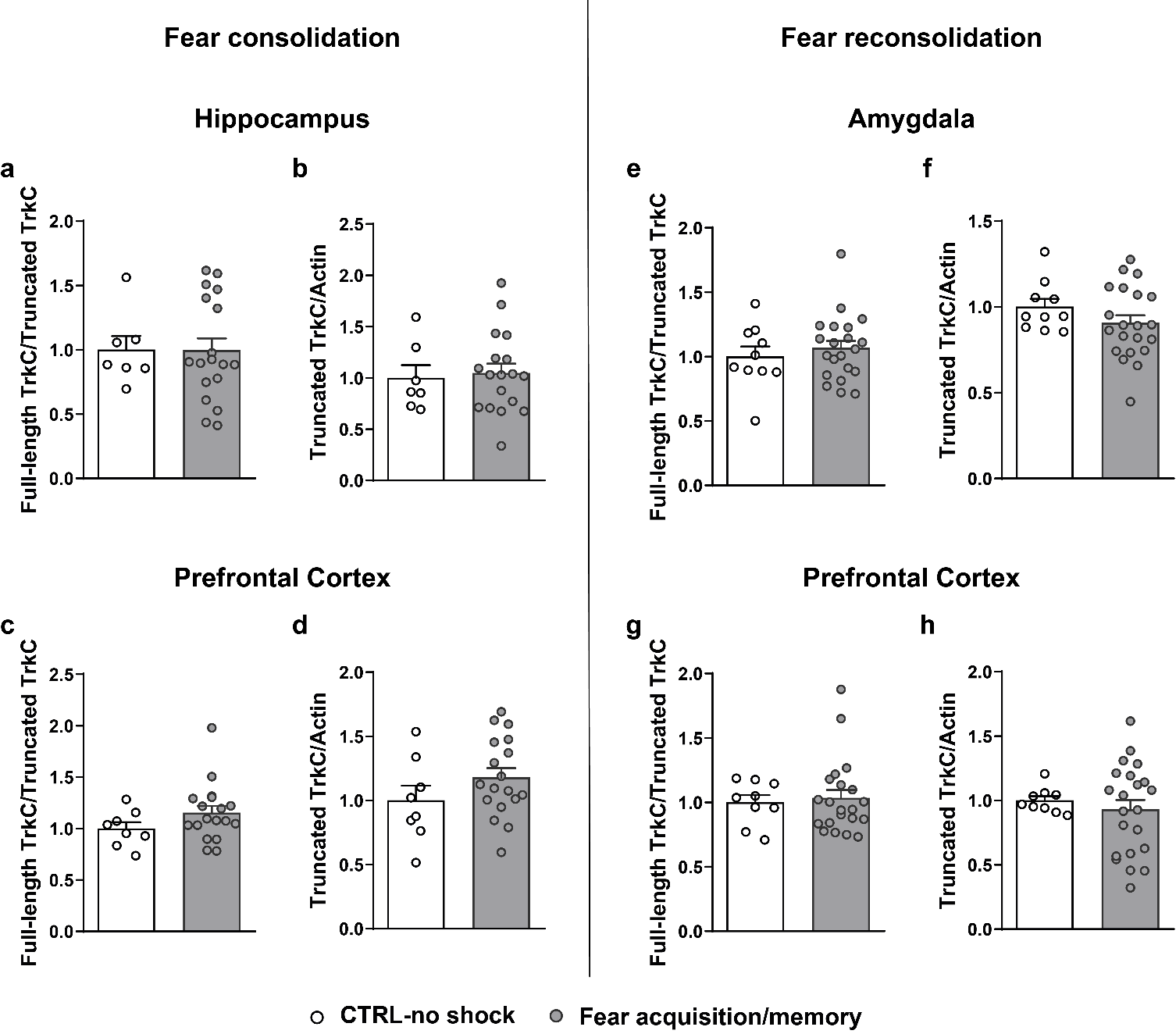


**Fig. S1** Expression levels of truncated TrkC in the brain fear circuit during contextual fear (re)consolidation. Quantification of **(a, c, e, g)** full-length/truncated TrkC ratio and **(b, d, f, h)** truncated TrkC levels in total protein extracts from **(a, b)** the hippocampus (fear acquisition n = 18; CTRL-no shock n = 7) and **(c, d)** the prefrontal cortex (fear acquisition n = 17; CTRL-no shock n = 7) of mice sacrificed during fear consolidation; and from **(e, f)** the amygdala (fear memory n = 21; CTRL-no shock n = 10) and **(g, h)** the prefrontal cortex (fear memory n = 21; CTRL-no shock n = 9) of mice sacrificed during fear reconsolidation. β-actin was used as a loading control. Representative western blot images showing full-length and truncated TrkC under CTRL-no shock and fear acquisition/memory conditions are shown in Fig. 1b, j and Fig. 2g, k. CTRL, control.
